# Mental health services and R&D in South Korea

**DOI:** 10.1186/s13033-016-0077-3

**Published:** 2016-06-02

**Authors:** Sungwon Roh, Sang-Uk Lee, Minah Soh, Vin Ryu, Hyunjin Kim, Jung Won Jang, Hee Young Lim, Mina Jeon, Jong-Ik Park, SungKu Choi, Kyooseob Ha

**Affiliations:** Department of Mental Health Research, Seoul National Hospital, Seoul, South Korea; Department of Psychiatry, Hanyang University School of Medicine, Seoul, South Korea; Department of Preventive Medicine, School of Medicine, Kyung Hee University, Seoul, South Korea; Department of Addiction Psychiatry, Seoul National Hospital, Seoul, South Korea; Department of Psychiatry, Seoul National Hospital, Seoul, South Korea; Division of Planning & Public Relations, Seoul National Hospital, Seoul, South Korea; Department of Psychiatry, Kangwon National University School of Medicine, Chunchon, Korea; Seoul National Hospital, 389 Neungdong-ro, Gwangjin-gu, Seoul, 04933 South Korea

**Keywords:** Mental health services, Mental health R&D

## Abstract

World Health Organization has asserted that mental illness is the greatest overriding burden of disease in the majority of developed countries, and that the socioeconomic burden of mental disease will exceed that of cancer and cardiovascular disorders in the future. The life-time prevalence rate for mental disorders in Korea is reported at 27.6 %, which means three out of 10 adults experience mental disorders more than once throughout their lifetime. Korea’s suicide rate has remained the highest among Organization for Economic Cooperation and Development (OECD) nations for 10 consecutive years, with 29.1 people out of every 100,000 having committed suicide. Nevertheless, a comprehensive study on the mental health services and the Research and Development (R&D) status in Korea is hard to find. Against this backdrop, this paper examines the mental health services and the R&D status in Korea, and examines their shortcomings and future direction. The paper discusses the mental health service system, budget and human resources, followed by the mental health R&D system and budget. And, by a comparison with other OECD countries, the areas for improvement are discussed and based on that, a future direction is suggested. This paper proposes three measures to realize mid and long-term mental health promotion services and to realize improvements in mental health R&D at the national level: first, establish a national mental health system; second, forecast demand for mental health; and third, secure and develop mental health professionals.

## Background

Mental health is recognized as an important global issue. The Organization for Economic Cooperation and Development (OECD) reported that the direct and indirect costs of mental health problems exceeded 4 % of the gross domestic product (GDP) on average [1]. World Health Organization (WHO) has asserted that mental illness is the greatest overriding burden of disease in the majority of developed countries, and that the socioeconomic burden of mental disease will exceed that of cancer and cardiovascular disorders in the future [2]. A study on the burden of non-communicable diseases revealed that the economic cost of mental diseases is USD16.3 trillion across the globe [3]. The life-time prevalence rate for mental diseases in Korea is reported at 27.6 %, meaning three out of 10 adults experience mental illness more than once throughout their lifetime [4]. Total medical expense related to mental diseases was USD1.05 billion as of 2013 [5], accounting for about 3.4 % of total medical expenses within the same year.

In particular, Korea’s suicide rate has remained the highest among OECD nations for 10 consecutive years with 29.1 people out of every 100,000 having committed suicide [6]. A study revealed that 75.3 % of those who had attempted suicide experienced more than one mental disorders [4], which highlights the importance of mental health in addressing serious social problems like suicide. The level of interest in mental health is growing in every corner of society.

The importance of interest in mental health is both on the rise not only in Korea but also internationally. WHO’s comprehensive mental health action plan in 2013 set a goal to increase management of the mentally ill by 20 % in each country by 2020 [7]. Efforts to develop new treatments through R&D investments and the adoption of new therapies in medical fields are necessary so as to improve mental health services. In this respect, the National Institute of Health (NIH) in the United States emphasized the importance of investment in translational research that facilitates the application of basic science research findings in medical practice [8].

A comprehensive study on mental health services in clinical practice and current status of R&D in Korea is hard to come by, which is suggestive of the difficulty to come in planning and implementing national mid and long-term mental health services and making improvements in R&D. Against this backdrop, this paper examines the current status of the mental health services and R&D in Korea, and discusses the associated problems and future direction of both.

## Mental health services in Korea

According to a WHO report, 68 % of the nations surveyed had mental health policies and plans, and 51 % enacted laws on mental health [9]. In Korea, mental health services made qualitative and quantitative advancements after the enactment of the Mental Health Act in 1995. The legislation had a significant influence in terms of shifting focus to community—based mental health services that underline rehabilitation and social restoration [10]. In 1998, the government established the ‘First Five Year Plan for National Mental Health Promotion’ in addition, the Amendment of the Mental Health Act included a regulation on the establishment of mental health services plans by central and local governments every 5 years, which enabled the consistent planning of mental health care at the national level [11].

### Mental health service system

**Fig. 1:**
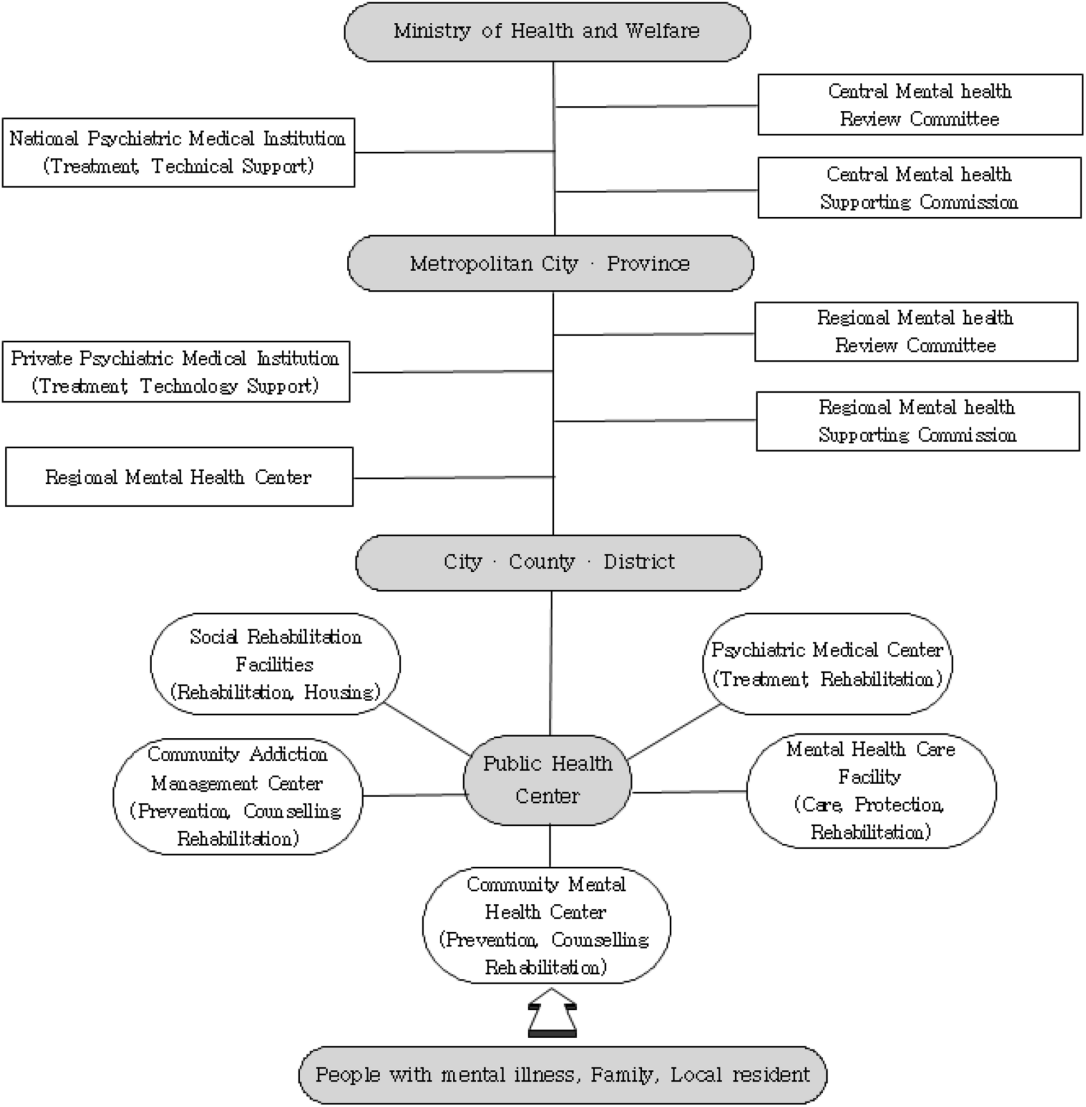
Mental health service delivery system [12]

Mental health promotion services in Korea are comprised of (1) the treatment of illnesses in medical institutions, (2) community-based psychosocial rehabilitation, and (3) housing, occupational and economic support. In detail, treatments in medical facilities refer to medical examinations, treatment and rehabilitation conducted by national/public and private psychiatric medical institutions. Community-based services refer to the management of the chronically ill and education for the general public for the purpose of promoting mental health and social rehabilitation facilities (housing support, day rehabilitation, etc.) [12]. Refer to Fig. 1 below.

According to the 2013 Mental Health Statistics [13], the number of psychiatric medical centers increased 1.65-fold from 822 to 1354, community rehabilitation centers grew 4.57-fold from 121 to 554, and psychiatric nursing homes rose 1.07-fold from 55 to 59 between 2001 and 2013. Psychiatric medical centers as of the end of 2013 included 187 mental hospitals (19 national/public, 168 private), 314 hospitals with a mental health unit (181 general hospitals, 133 hospitals), and 853 psychiatric clinics. In addition, community rehabilitation centers as of the end of 2013 included 304 social rehabilitation facilities, 200 mental health centers (11 regional, 189 local), and 50 Community Addiction Management Centers (Table 1). As of 2013, the number of people using services offered by community rehabilitation centers was 79,379 (excluding registered children/adolescents) and the number of children/adolescents using mental health centers was 15,608. In comparison with the number of mental health facilities per 100,000 population in other countries such as Japan, UK and the US, there were more number of mental health outpatient facilities (Korea = 2.35, Japan = 2.31, USA = 1.95, UK = 4.94) and beds in mental hospitals in Korea (Korea = 149.23, Japan = 204.4, USA = 19.44, UK = 7.99) whereas there are less community residential facilities (Korea = 0.26, Japan = 1.57, USA = 0.65, UK = 67.8) [14]. The number of mental health facilities seems to influence a patient’s actual use of mental health services. Given the fact the life-time prevalence rate for mental disorders in Korea is reported at 27.6 % [4], the number of people using mental health hospitals as of 2014 was 3869 people per 100,000 population showing that approximately 14 % of people utilized the mental health hospitals [15, 16]. In case of community residential facilities, 189 people per 100,000 population are using the services as of 2016 [16, 17]. This represents that only 0.68 % of people used the community residential facilities. Therefore, this indicates that most mental health patients in Korea receive a hospital-based mental health treatment rather than a community-based treatment.

**Table 1:** Number of mental health facilities across the nation [13]

Classification	2001 (A)	2005	2010	2013 (B)	B/A
Psychiatric Medical Center					
Mental hospital					
National/public	17	18	18	19	1.11
Private	57	63	127	168	2.94
Mental Health Unit in Hospital					
General Hospital	154	168	165	181	1.17
Hospital	53	81	136	133	2.50
Psychiatric clinic	541	718	827	853	1.57
Sub-total	822	1048	1273	1354	1.65
Community Rehabilitation Center					
Social Rehabilitation Facility	66	138	230	304	4.60
Mental Health Center					
Regional^a^	–	–	–	11	N/A
Local	46	62	157	189	4.10
Community Addiction Management Center	9	20	41	50	5.55
Sub-total	121	220	428	554	4.57
Psychiatric Nursing Home	55	56	59	59	1.07
Total	998	1324	1760	1967	1.97

^a^Data on regional mental health centers was collected as of the end of 2013 only

The National Mental Health Five-Year Plan (2011–2015) classifies mental health services into the following six categories: first, reduce inappropriate hospital stays for patients with serious mental illness, and promote social return and participation; second, manage the quality of treatments in medical centers and community-based services; third, prevent and improve mental disorders in various groups; fourth, establish a system to manage alcohol use disorder (AUD) owing to the sociocultural characteristics of Korean; fifth, decrease social prejudice towards mental disorders and protect the human rights of individuals with mental illness; and sixth, build an infrastructure, provide access to services and manage human resources [18]. Importance seems to be placed on the provision of evidence-based services and quality enhancement, the need to intervene in the mental health of children and the elderly, and the professionalism of mental health experts as regards to the ability to provide adequate services. Services are provided based on the following four life-cycle stages: infant and toddler (0–6), children and adolescents (7–18), young and middle-aged adult (19–64), and old age (65 and older). Considering the number of mental health hospitals (*N* = 1354) and mental health services funded by government (*N* = 4018) [19–37], children and adolescents are able to use 89.2 % of total services. In case of middle-aged and old-aged adults, they can utilize 48.7 and 41.6 % of total services respectively. Given the fact that services were not clearly divided into each age group, the percentage of the services was not 100 %. In addition, services are provided depending on the types of mental health problems such as psychiatric disorder, addiction, suicide, and mental wellbeing. The treated prevalence of psychiatric disorders is assumed to be 1511 people per 100,000 population [15, 16] and the number of mental health hospitals and services funded by government and specialized for psychiatric disorders is approximately 2064 [19, 22]. Moreover, the treated prevalence of addiction is estimated as 171 people per 100,000 population [15, 16] and the number of mental health hospital and services funded by government and specialized for addictions is approximately 184 [19, 31–36, 38]. In case of suicide, it has been reported that as of 2011, the lifetime prevalence of suicide ideation was 15.2 %, suicide plan was 3.3 % and suicide attempt was 3.2 % [39]. And, the actual number of suicide completer was 27.3 people per 100,000 population as of 2014 [40]. Government provides mental health services for suicide attempters through mental health centers and suicide prevention centers (233 centers), and emergency services at 25 hospitals [41]. The number of mental health services to improve mental wellbeing of general population is approximately estimated to be 773 [20, 21, 23–26, 37]. Based on the mental health intervention spectrum, there are three levels of prevention: primary prevention, secondary prevention (treatment), and tertiary prevention (rehabilitation). Research found that all of the relevant government organizations apart from the Ministry of Health & Welfare focus on primary prevention [42].

### The budget for mental health services

Mental health services in Korea are divided into the following three categories depending on their source of financing: first, the operation of mental health care facilities and social rehabilitation facilities supported by local governments (50 % of national subsidies being limited to functional reinforcement projects only); second, community-based mental health promotion services including mental health centers supported by local and central governments (50 % each); and third, improvements in mental illness awareness, the evaluation of mental health institutions, and the operation of Korea Suicide Prevention Center, the installation and operation of which are supported by the central government. The central government’s budget for mental health services more than doubled in the past 5 years to about USD 18 million in 2010, USD 21 million in 2011, USD 30 million in 2012, USD 38 million in 2013, and USD 43 million in 2014. Nonetheless, the mental health budget compared to the total health care expenditures was relatively low, being only 2.6 % of the total expenditures as of 2014 [43].

A breakdown of the budget by programs, which is presented in Table 2, includes USD 6.7 million for mental health promotion pilot programs, USD 6.6 million for suicide prevention, USD 4.5 million for the prevention and management of addictions, USD 2.9 million for children/adolescents mental health promotion, USD 1.9 million for mental health promotion, and USD 0.2 million for the removal of the social stigma against mental illness and to improve public awareness. The budget for suicide prevention had the highest growth rate, increasing tenfold since 2010 [43].

**Table 2:** Central Government Budget for Mental Health Promotion [43]

Classification	2010	2011	2012	2013	2014
Establish/operate regional/local mental health center	9946 (54.4)	10,593 (50.2)	12,340 (41.1)	13,684 (36.5)	15,577 (35.9)
Mental health promotion services for children/adolescents	921 (5.04)	921 (4.36)	922 (3.07)	2194 (5.85)	2852 (6.57)
Mental health promotion services^a^	0	0	0	1492 (3.98)	1878 (4.33)
Prevention and management of addiction^b^	3660 (20.0)	3869 (18.3)	4252 (14.2)	4500 (12.0)	4483 (10.3)
Prevention of suicide^c^	645 (3.53)	1259 (5.96)	2001 (6.67)	4195 (11.2)	6618 (15.3)
Support mental health facilities^d^	2782 (15.2)	3721 (17.6)	3820 (12.7)	3820 (10.2)	3438 (7.92)
Removal of prejudice towards mental disorders and improving public awareness	281 (1.54)	281 (1.33)	267 (0.89)	260 (0.69)	242 (0.56)
Evaluate psychiatric medical centers	0	0	244 (0.81)	895 (2.39)	1155 (2.66)
Pilot program on comprehensive mental health promotion^e^	0	0	5936 (19.8)	5936 (15.8)	6713 (15.5)
Others^f^	39 (0.21)	470 (2.23)	256 (0.85)	529 (1.41)	438 (1.01)
Total	18,274 (100)	21,115 (100)	30,037 (100)	37,505 (100)	43,394 (100)

Unit: USD 1 thousand

Exchange rate on 22 April 2016, 1 Dollar = 1139.40 Won

^a^Follow-up on mental health screening, promote mental health of public social workers

^b^Establish/operate Community Addiction Management Center, manage addiction cases including the homeless, treat and protect people with narcotics addiction, prevent alcohol-related problems

^c^Establish/operate Korea Suicide Prevention Center, suicide prevention services and community suicide prevention services

^d^Establish/operate mental health care facilities and support their functional reinforcement

^e^Gwangju Trauma Center, and establish center for integration

^f^Other costs related to mental health promotion services

### Human resources in mental health services

Human resources in mental health services in Korea include psychiatrists, mental health professionals and non-professionals. Both mental health professionals and non-professionals are comprised of nurses, social workers and psychologists. However, mental health professionals are those who receive specialized mental health training. In case of mental health nurses and social workers, they should have at least one-year training after they are qualified as a nurse or graduated with social work degree [44, 45]. And, to become clinical psychologists, they should have a master degree in clinical psychology and gain at least 3 years training [46]. By employment status, the number of full-time employees in nation-wide psychiatric health facilities, was 20,175 as of the end of 2013 (Table 3). By facility, 16,175 people worked full-time in psychiatric medical centers, accounting for 80 % of the total, followed by 1093 (5.4 %) people in psychiatric nursing homes and 2907 (14.4 %) people in community rehabilitation facilities. Community rehabilitation facilities with a greater percentage of professionals and non-professionals had 1740 of the former and 1152 of the latter. Some 1740 professionals include 556 nurses (32.0 %), 1070 social workers (61.5 %), and 114 clinical psychologists (6.5 %), while some 1152 non-professionals include 229 nurses (18.2 %), 923 social workers (80.1 %), and 20 clinical psychologists (1.7 %). Community rehabilitation facilities had a relatively few full-time psychiatrists and other medical doctors, which is considered to be due to the fact that a number of psychiatrists are able to work at community rehabilitation facilities for at least 16 h in a week whilst having a main job at different institutions [19].

**Table 3:** Human Resource in Nationwide Psychiatric Health Facilities (2013) [13]

Classification	Psychiatrist	Psychiatric resident	Mental health professional	Non professional and others	Total
Psychiatric Medical Center					
Mental hospitals	840	146	1287	4427	6700
(General) hospital Mental health unit	994	536	1016	3465	6011
Psychiatric clinic	1080	33	197	2154	3464
Sub-total	2914	715	2500	10,046	16,175
Mental Health Care Facilities	49	–	69	975	1093
Community Rehabilitation Center					
Regional Mental Health Center	1	–	163	20	184
Local Mental Health Center	12	–	930	532	1474
Social Rehabilitation Facility	1	–	526	509	1036
Community Addiction Management Center	1	–	121	91	213
Sub-total	15	–	1740	1152	2907
Total	2978	715	4240	12,173	20,175

Unit: person

## Mental health R&D in Korea

WHO underlined the importance of R&D [47] to improve global health and health equity [18]. Korea began its investment in mental health R&D at the national level based on the Brain Research Promotion Act enacted in 1998. A significant portion of the investment finances brain research while covering mental health related R&D as well [48].

### The mental health R&D system

**Fig. 2:**
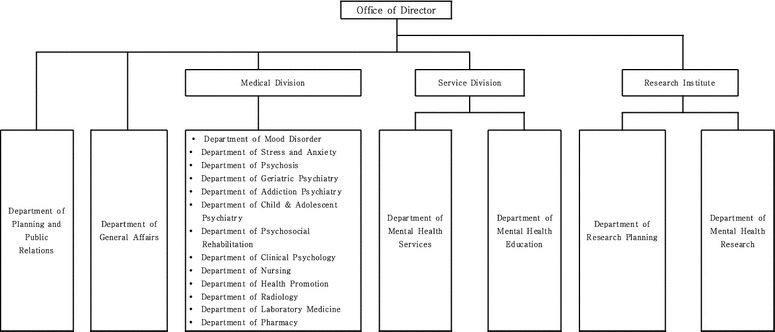
Structure of National Center for Mental Health

Korea does not have a separate national (public) research institution dedicated to mental health research. However, there are two major institutions that produce studies related to mental health: the National Mental Hospital that studies mental health promotion and prevention, and the Korea Brain Research Institute that studies brain diseases [48]. The National Mental Hospital focuses on treatment and management, hence there are plans to establish a National Center for Mental Health as an independent entity to strengthen mental health R&D activities. The center is expected to conduct research on national mental health policy, treatment, and intervention. Its organizational structure is described in Fig. 2 below. To be more specific, the center will include the Division of Medicine, the Division of Mental Health Service and the National Institute of Mental Health. The Division of Medicine consists of 13 departments including the department of psychiatry, the department of mood disorders, department of stress and anxiety, department of psychosis, department of geriatric psychiatry and so on. The Division of Mental Health Services will be in charge of public mental health services as well as mental health education and the National Institute of Mental Health will undertake research planning and mental health research.

**Fig. 3:**
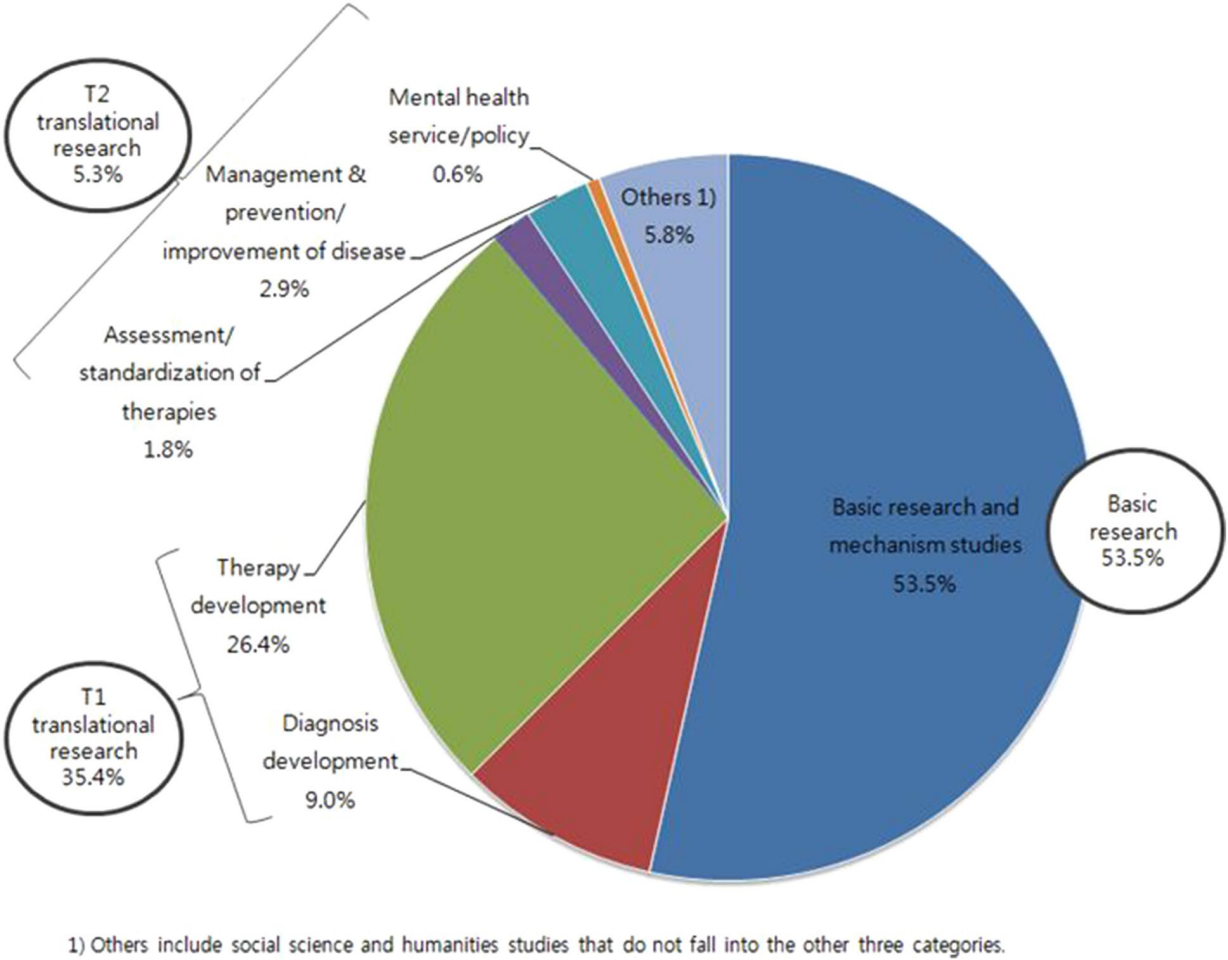
Annual average investment in National Mental Health R&D in 2008–2012 [50]

Mental health R&D in Korea can be divided into basic-translational-development in accordance with the Health Research Classification System in the UK. The first stage of translational research (T1) transfers findings from basic research to clinical drugs, medical instruments, diagnosis and treatment. The second stage of translational research (T2) transfers the research achievements to medical fields to improve health care performance [49]. It has been reported that investments in Korea is centered on T1 translational research. As a result, Korea lags behind in investments in T2 translational research that enhances the effect of evidence-based mental health policies. Investment in national mental health R&D between 2008 and 2012 can be broken down into the following four categories, which is presented in Fig. 3; about 53.5 % of the investments went into basic research and mechanism studies, about 35.4 % went into T1 translational research (develop therapy and diagnosis), about 5.3 % went into T2 translational research (policy on mental health services, disease management and prevention, assessment and standardization of therapies), and about 5.8 % went into others (humanities and social science researches, etc.). This allocation of investment in national mental health R&D represents that mental health R&D in Korea still hugely focuses on medical approach to mental healthcare and lags behind the development of T2 research. On the other hand, other countries such as UK and the US put large investigations on T2 [50]. According to the UK Health Research Analysis (2012), mental health R&D funding on T2 is about 39 % of total R&D which is about 5 times larger than funding on T1 [50].

### The budget for mental health R&D

**Fig. 4:**
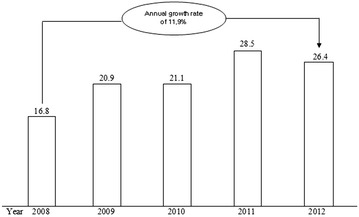
Changes in National Mental Health R&D Investment (USD 1 million) [50]. Exchange rate on 22 April 2016, 1 Dollar = 1139.40 Won

According to the Ministry of Health and Welfare (MOHW) [50], the central government is the primary source of financing for mental health R&D. MOHW reports that investment in R&D in 2008 was USD16.8 million, which increased at an annual rate of 11.9 % to about USD26.4 million in 2012 (Fig. 4). However, the proportion of R&D investment in overall health care and medical expenditures continues to decline. By government entities, the Ministry of Education & Science Technology invested USD17.3 million (63 %), the Ministry of Health & Welfare invested USD6.41 million (24 %), the Ministry of Knowledge Economy invested USD1.67 million (6 %), the Ministry of Food and Drug Safety invested USD0.79 million (3 %), and the small and medium business administration invested USD0.70 million (3 %). The categorization of investments by mental health R&D projects as of 2012 includes investment of USD4.01 million in depression, USD2.98 million in schizophrenia, USD1.40 million in suicide, USD1.18 million in autism, USD1.17 million in behavioral addiction, USD1.10 million in drug addiction, USD0.93 million in ADHD, USD0.90 million in bipolar disorder, USD0.53 million in anxiety disorder, and USD0.50 million in developmental disability.

## Suggestions for the future direction

### Areas of improvement for mental health services and R&D in Korea

#### Mental health services

A study revealed that in Korea, 15.3 % of those afflicted with mental illnesses received mental health services. In addition, it is reported that 23 % of those with depression are treated in Korea, which is far less than the 44 % rate of treatment in other developed countries [4]. An analysis of mental health services in Korea identified a high tendency to utilize repetition of services depending on the service recipients and the overlapping of services for behavioral addiction [42].

World Health Organization recommends that the mental health budget takes up 50~15 % of health care expenditures. However, an investigation among OECD nations showed that the rate in Korea stands at an average of 3 %, falling short of meeting the WHO recommended level [51]. Furthermore, the National Health Promotion Fund (subject to change depending on annual cigarette sales as the fund is financed through tobacco taxes) finances a significant portion of the mental health budget, which suggests there will be difficulty securing future budgetary allotments [52].

Moreover, since mental health services are interactive, it is important to have professional experts to guarantee quality service [53]. Among OECD countries, Korea has 5.12 psychiatrists (19th), 21.61 mental health nurses (18th), 1.12 mental health clinical psychologists (23rd) and 6.01 mental health social workers (19th) per 0.1 million people [54]. Furthermore, WHO members have an average of nine mental health professionals per 0.1 million people [9], whereas the median value of the community mental health resources (province) in Korea is only 2.9 per 0.1 million people [13].

#### Mental health R&D

Unlike Korea, the US and Japan have organizations dedicated to mental health R&D. In the US, three research institutes including the National Institute of Mental Health (NIMH), the National Institute on Alcohol Abuse and Alcoholism, and the National Institute on Drug Abuse (NIDA) under the National Institutes of Health perform public mental health R&D activities. In Japan, the Ministry of Health, Labour and Welfare operates the National Center of Neurology and Psychiatry specialized in mental health and neurological disorders [55].

In the US and the UK, governments take the initiative to make investments in R&D projects so as to address mental health issues that are expected to be the greatest overriding burden of diseases in the future. Out of the total of national mental health R&D investments, the US spends 21.3 % in T2 translational research while the UK spends 39 %. However, in Korea, only 5–6 % of the total investment goes to T2 translational research [50].

Jacob et al. [56] have looked at the mental health R&D activities in 37 countries including Korea and found that 22 countries were monitoring mental health service data. However, due to a scarcity of data, it is difficult to identify types of research and the number of mental health research professionals participating in the research [56]. Only available data is a total number of researchers involved in R&D which was 410,333 staffs as of 2013 [57]. However, the fields of R&D that staffs participated in were unknown. Therefore, it is difficult to know how many researchers were involved in mental health R&D. And, only 1.8 % of the national health promotion R&D investment is allocated to human resource development [58].

### The future direction of mental health services and R&D

Efforts to overcome the aforementioned challenges and the consideration of the future direction are required in order to improve mental health services and R&D in Korea. These goals are not achievable in a short period of time. Like many other national plans, a mid and long-term plan of 5–10 years must be in place. From this perspective, suggestions to provide mid and long-term national mental health promotion services and to enhance mental health R&D include the following:

First, establish a national mental health system.

With no independent organization that supervises mental health R&D, it is difficult to implement systematic and comprehensive management of R&D activities in Korea. It is safe to say that most investments in mental health research projects have been sporadic and have lacked continuity. Therefore, an effective measure would be to establish a national mental health center with clearly defined roles and responsibilities. In the US, NIMH and NIDA focus on research while the Substance Abuse and Mental Health Services Administration takes charge of national mental health governance. Additionally, in Japan, the National Center of Neurology and Psychiatry focuses on research for mental health and neurological disorder [55]. However, such institutions are not existed in Korea. Therefore, the national mental health center scheduled to be established will need to manage and support both the mental health policy programs and research to produce scientific evidence.

Second, forecast future demand for mental health services.

The mental health budget in Korea continues to rise. Nevertheless, there is much room for improvement when compared to other nations or when considering the portion of the mental health budget out of total health care expenditures. As of 2015, the mental health budget in Korea was only 2.6 % of total health budget [59]. However, this proportion of budget is smaller than mental health funding in UK, even comparing with the result of 2011 (10.8 %) [60]. Although mental health R&D should be conducted to forecast future demand for mental health services, only small budget is allocated in the mental health R&D as well. According to the NIH funding on R&D as of 2012 in the US, the mental health R&D funding constitutes approximately 9.8 % of total funding on R&D [61]. In contrast, the mental health R&D funding in Korea only accounts for 2.7 % of total funding on R&D [61]. Therefore, additional budgetary allotments should be obtained to improve R&D and mental health services. However, it is not easy to anticipate the additional budgetary while preparing projects. Hence, it is necessary to forecast future demand to set priorities and select tasks, based on which strategic investments in mental health services and R&D are made within the limited budget.

Third, secure and develop professional experts

Korea faces serious mental health professional resource issues not only in terms of services but also in R&D. As for the service issues, the problem lies in the high turnover and retirement rates that result from professionals being overburdened by unlimited workloads. Since there is no guideline for user and staff ratio in mental health centers, one staff is taking care of 100 cases in some centers [62]. Therefore, it is necessary to set up a suitable user and staff ratio. In case of assertive outreach teams in the UK, the Mental Health Policy Implementation Guide (MHPIG) set up an ideal users and staff ratio as 10:1 [63]. Moreover, poor job security seems to be another factor contributing to the high turnover and retirement rates. In fact, the most of mental health professionals working in mental health centers are employed on fixed-term contracts [62]. Therefore, it is necessary to increase permanent job positions in mental health centers through a government policy. As for the R&D issues, the problem arises from lack of infrastructure and system that can nurture human resources. In the US, the importance of investigating on researcher development is emphasized in order to maintain competitiveness in mental health R&D [61]. Likewise, there needs to be a consideration for the establishment of a national system and infrastructure to develop human resources.

## Conclusions

This paper examined the overall status of mental health services and R&D in Korea. The system, budget, and human resources of mental health services were described followed by a description of the system and budget for R&D. Following that, comparisons with other countries were made to identify areas for improvement, and to find suggestions for future direction to improve mental health services and R&D in Korea. The paper concludes by recommending implementation of three measures to provide mid and long-term mental health promotion services at the national level and to improve mental health R&D. The measures include first, building a national mental health system, second, forecasting mental health demand, and third, securing and developing mental health professionals.

## Abbreviations

WHO: World Health Organization
OECD: Organization for Economic Cooperation and Development
R&D: Research and Development
GDP: gross domestic product
USD: United States Dollars
NIH: National Institute of Health
AUD: alcohol use disorder
T1: first stage of translational research
T2: second stage of translational research
MOHW: Ministry of Health and Welfare
KRW: Korean Won
NIMH: National Institute of Mental Health
NIDA: National Institute of Drug Abuse
